# Dermal Matrix Fixation: A Good Adhesion to Wound Edges without Vascularization Impairment

**DOI:** 10.1097/GOX.0000000000002327

**Published:** 2019-07-24

**Authors:** Marta Starnoni, Giorgio De Santis, Massimo Pinelli

**Affiliations:** From the Division of Plastic Surgery, University of Modena and Reggio Emilia, Modena, Italy.

Acellular matrix products are used to treat partial and full thickness wounds of different origin (pressure ulcers, venous leg ulcers, surgical wounds, chronic vascular ulcers, second-degree burns, and draining wounds).

Dermal regeneration matrix (Integra LifeSciences, Plainsboro, N.J.) is a synthetic scaffold that acts as a bilayered skin regeneration template.^1^ The top layer simulates the epidermis and consists of a semipermeable polysiloxane (silicone) that limits bacterial invasion and moisture absorption. The bottom layer consists of a cross-linked bovine tendon collagen and glycosaminoglycan that promotes cell migration and induces regeneration of the dermis. It is a biodegradable matrix that acts as a scaffold for fibroblast and endothelial invasion and capillary growth from the surrounding wound edges.

Skin transplantation can be performed only if the dermal matrix demonstrates a good in-take, with no signs of infection, and is well anchored in the wound. Therefore, connection of the wound bed to the matrix is crucial for graft and template survival.^1^

Integra can be anchored in different ways: simple suture, horizontal or vertical mattress sutures, Allgower-Donati sutures, staples, or fibrin glue.

Fibrin glue layer may be too occlusive, preventing cell migration and degradation of the scaffold.^2^

A senior surgeon was asked to evaluate the technical aspects of fixation of dermal matrix to wounds (of different origin, location, and size) performed by 2 residents. In particular, 4 groups of 20 patients each: group A (simple sutures), group B (horizontal or vertical mattress sutures), group C (Allgower-Donati sutures), and group D (staples). Whereas groups A, B, and D were evaluated as good fixation, group C was evaluated as excellent fixation.

**Fig. 1.**
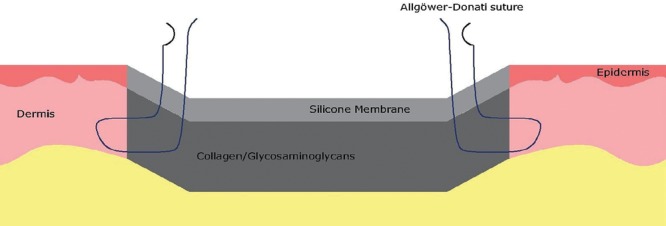
Illustration of the fixation of dermal matrix to a full thickness skin defect with Allgower-Donati sutures.

In our opinion, this excellent result in group C could be explained because of the good adhesion of the wound edges to the dermal matrix without kinking or folding of the skin. In fact, Allgower-Donati sutures pull the dermal matrix into opposition with the side of the wound, without skin kinking or folding (Fig. 1). The other suture patterns show bunching or folding of the skin. This folding might result in vessels deformation (such as kinking) or stretching of the blood vessels suppling the dermis, thereby decreasing the cutaneous blood flow.^3^

Simple, vertical, and horizontal mattress sutures are designed to evert skin edges and can allow a good adhesion between the dermal matrix and the wound edge but they cause a deformation of the skin with impairment of vascularization. Staples do not allow a good adhesion and although care is taken not to tightly suture the clips, they constitute a massive technique of closure that can cause ischemia of the embraced tissue. The Allgower-Donati suture is passed in the intradermal layer and leaves the microcirculation to the skin and the wound edge intact.

Decision-making regarding closure technique is based on surgeons’ subjective experience, but it has been demonstrated that the Allgower-Donati suture had the smallest effect on cutaneous blood flow compared with vertical mattress, horizontal mattress, and simple suture.^3,4^ In our opinion, these concepts could be applied to dermal matrix fixation.
